# Editorial: Follicle-stimulating hormone: Fertility and beyond-volume II

**DOI:** 10.3389/fendo.2022.991106

**Published:** 2022-08-03

**Authors:** Manuela Simoni, Ilpo Huhtaniemi, Livio Casarini, Daniele Santi

**Affiliations:** ^1^ Unit of Endocrinology, Department of Biomedical, Metabolic and Neural Sciences, University of Modena and Reggio Emilia, Modena, Italy; ^2^ Department of Surgery and Cancer, Institute of Reproductive and Developmental Biology, Imperial College London, London, United Kingdom

**Keywords:** FSH (follicle stimulating hormone), fertility, FSHR (follicle-stimulating hormone receptor), polymorphism, gonadotropins

This Research Topic extends the scientific coverage of the one previously published and dedicated to follicle–stimulating hormone (FSH) and its receptor (FSHR), which raised great interest among scientists in the field. FSH is a gonadotropin used in clinical practice on the one hand for the treatment of hypogonadism, on the other for infertility. In particular, FSH is used in females during assisted reproduction to stimulate multifollicular growth and ovulation and in males to boost spermatogenesis. This increasing FSH use in clinical practice favored the development of several biosimilar molecules reaching recently the market. Biosimilars and their originators (correct word)? show different glycosylation patterns, an issue worthy of further investigation. The identity and the clinical impact of the glycan structures bound to the molecule are still unclear. Similarly, the effect of an individual’s genotype on the response to FSH treatment still remains confusing. These variables are fundamental in the personalization of pharmacological approaches in assisted reproduction, although this application is still ambiguous, challenging, and incompletely understood. Further steps forward must be taken to reach full view of the mode of FSH action and to understand the role of genetics and environment in modulating the gonadal response to this hormone.

Scientists in the field contributed to the elucidation of clinical and pathophysiological implication related to FSH. A collection of six research articles and two reviews deepens the role of glycosylation in FSH function, the use of the hormone in women and men undergoing assisted reproduction with different clinical pictures, new aspects of FSH intracellular signalling in endometrial cancer cells and the impact of endocrine disruptors.

## New human follitropin preparations: How glycan structural differences may affect biochemical and biological function and clinical effect

Dias and Ulloa-Aguirre comprehensively reviewed the complexity of FSH glycosidic forms, differentiating recently developed and commercially available molecules used for clinical treatments of infertility in humans. There is limited knowledge in this field since most of the studies available are from engineered non-gonadal host cells expressing recombinant receptors or animal models far to be fully representative of human physiology. The authors discussed how structural differences in glycosylation are related to specific biological functions and clinical effects of these drugs, such as pharmacokinetics and pharmacodynamics, and the ovarian response to controlled stimulation protocols.

## Human FSH glycoform α-subunit asparagine 52 glycans: Major glycan structural consistency, minor glycan variation in abundance

Bousfield et al. extended the overview on the functionally significant glycosylation variants of FSH. The study relies on an extensive molecular characterization of the multi-antennary nature of the glycan structures linked to hypo- FSH21 and fully-glycosylated FSH24 isoforms by mass-spectrometry. Structure-function relationships impacting the binding capacity and receptor assembly into multimers were pinpointed as the most intriguing and still unexplored issues of this gonadotropin’s physiology. The comprehension of these mechanisms provides an outlook into new strategies for personalized fertility treatments.

## Differential FSH glycosylation modulates FSHR oligomerization and subsequent cAMP signalling

The modulation of FSHR oligomerization and the impact on cAMP signalling of hypo- (FSH21/18) and fully-glycosylated (FSH24) hormone variants were deepened in the research article of Agwuegbo et al. FSHR complexes were analyzed in transfected cell models using a modified super-resolution imaging technique (PD-PALM) upon treatment with different FSH glycoforms. The authors demonstrated how receptor-receptor assemblies would be linked to glycosylation and modulate the kinetics of cAMP production, suggesting specific targeting of FSHR as a potential and novel therapeutic approach to assisted reproduction.

## Recombinant human follicle-stimulating hormone alfa dose adjustment in US clinical practice: An observational, retrospective analysis of a real-world electronic medical records database

Mahony et al. performed an observational, retrospective analysis of data from an electronic de-identified medical records database including 39 clinics in the USA to evaluate FSH dose adjustment during ovarian stimulation for assisted reproduction. This real-world data analysis highlighted that dose adjustment is common in clinical practice in the USA. In particular, dose adjustment was more frequent in younger than older women, as well as in those with a high versus sub ovarian reserve or those with ovulation disorders and polycystic ovary syndrome. The authors described how the personalization of the FSH administration is applied in real clinical practice.

## Effects of FSHR and FSHB variants on hormonal profile and reproductive outcomes of infertile women with endometriosis

A cross-sectional study was performed on 213 infertile Brazilian women with endometriosis and undergoing assisted reproduction, aiming to evaluate the effects of the *FSHB*:c.-211G>T, *FSHR*:c.919G>A, and *FSHR*:c.2039G>A polymorphisms on reproduction outcomes (Bianco et al.). The authors’ contribution demonstrated the role of *FSHB* and *FSHR* gene polymorphisms on both hormonal profile and assisted reproduction outcomes in women with endometriosis treated in assisted reproduction.

## FSHB genotype identified as a relevant diagnostic parameter revealed by cluster analysis of men with idiopathic infertility

A retrospective cohort study was performed to determine whether idiopathic male infertility could be sub-grouped by an unbiased clustering approach and whether underlying etiologic factors could be delineated (Krenz et al.). Common andrological features, such as anthropological, semen and hormonal parameters were included, including the *FSHB* c.-211G>T (rs10835638) single nucleotide polymorphism. This is the first unbiased approach identifying sub-groups of idiopathic infertile men, revealing *FSHB* c.-211G>T polymorphism, FSH, and bi-testicular volume as strongest markers. This study confirmed the potential role of *FSHB* genotyping in the diagnostic routine of patients with idiopathic infertility.

## Endocrine disruption of the FSHR signalling during the human antral follicle growth

In this article, Roy et al. reviewed the insufficiently explored issue of the the impact of endocrine disruption on FSH-dependent reproductive functions. They provided an overview on the environmental pollutants with potentially direct modulating effects on FSHR expression, including their function and related intracellular signalling cascades. Disruptors have different modes of action, mainly grouped into agonists, antagonists or inverse agonists. Compounds indirectly impacting the action of FSH and its receptor were identified as well, providing a wide picture on the interactions between these molecules and the female reproductive system. Given the increasing involvement of environmental pollutants in human activities, the issue is particularly topical.

## FSH induces lipid droplets *via* gαi/o and β-arrestin in an endometrial cancer cell line

A novel mechanism of FSH-mediated signalling and downstream function was described in an endometrial adenocarcinoma cell line in the original *in vitro* research report from Sayers et al. The article describes a comprehensive cell signalling study, revealing how FSH induces the formation of Gαi protein/β arrestins-dependent lipid droplets in the absence of cAMP accumulation. This is a new mechanism of FSH action which could occur in specific FSHR-expressing adenoma/adenocarcinoma tumors. The data were matched with the detection of varying levels of FSHR, Gαi subtypes, and β arrestin by RNAseq data analysis of endometrial tissues, providing a novel mechanism to be exploited in personalized therapeutic approaches.

## Concluding remarks

The Associate Editors sincerely thank all authors for their excellent contributions to this Research Topic of Frontiers in Endocrinology. We feel that our efforts resulted in the coverage of the most recent research trends in FSH pathophysiology and genetics, deepening less well-known aspects, such as the clinical impact of hormone glycosylation, receptor oligomerization, modes of hormone action and the impact of environmental pollutants. Most importantly, we are confident that the new aspects of FSH/FSHR functioning compiled here will inspire future research and clinical approaches to inprover human fertility and combat cancer.

## Author contributions

MS, IH, LC and DS collaborated editing manuscripts published in the Research Topic. All authors contributed to the article and approved the submitted version.

## Conflict of interest

The authors declare that the research was conducted in the absence of any commercial or financial relationships that could be construed as a potential conflict of interest.

## Publisher’s note

All claims expressed in this article are solely those of the authors and do not necessarily represent those of their affiliated organizations, or those of the publisher, the editors and the reviewers. Any product that may be evaluated in this article, or claim that may be made by its manufacturer, is not guaranteed or endorsed by the publisher.

